# Phytochemical and antidiabetic evaluation of *Bauhinia tomentosa* L. aerial parts

**DOI:** 10.1186/s12906-026-05484-2

**Published:** 2026-08-01

**Authors:** Lourin G. Malak, Mai A. M. Ahmed, Ereny M. Abdelmalek, Rofida Wahman, Mariam A. Nicola, Adel A. Gomaa

**Affiliations:** 1 Department of Pharmacognosy, Faculty of Pharmacy, Assiut University, Assiut, 71526 Egypt; 2 Department of Pharmacology and Toxicology, Faculty of Pharmacy, Assiut University, Assiut, 71526 Egypt; 3 Faculty of Pharmacy and Pharmaceutical Research, Assiut National University, New Assiut City, Assiut 11511 Egypt; 4 Department of Pharmacognosy, Faculty of Pharmacy, Sphinx University, New Assiut City, Assiut 71515 Egypt; 5 Pharmacognosy Department, Faculty of Pharmacy, Badr University in Assiut BUA, Assiut, 77771 Egypt; 6 Department of Pharmacology, Faculty of Medicine, Assiut University, Assiut, Egypt; 7 Department of Medical Pharmacology, Faculty of Medicine, Assiut National University, New Assiut City, Assiut 11511 Egypt

**Keywords:** LC-QTOF-MS/MS, *Bauhinia*, Type 2 diabetes, Insulinotropic

## Abstract

**Background:**

Members of the genus *Bauhinia* L., commonly known as cow’s hoof, are important species in the family Fabaceae and are traditionally used to treat diabetes and other diseases. This study aimed to identify antidiabetic bioactive compounds from *Bauhinia tomentosa* aerial parts via bioassay-directed fractionation.

**Methods:**

Determination of serum glucose and insulin levels was conducted on nicotinamide + STZ-induced diabetic rats treated with different fractions via a glucose oxidase assay kit and a rat insulin ELISA kit. In addition, GLUT4 protein levels in muscle homogenates were quantified via an ELISA kit.

The ethyl acetate (EtOAc) fraction (the most active fraction) was fractionated via column chromatography (CC), and the isolated compounds were identified on the basis of their spectroscopic data. Additionally, the EtOAc fraction was characterized via LC‒QTOF‒MS/MS analysis. All the isolated compounds were evaluated for their antioxidant, *α*-glucosidase, and *α*-amylase inhibitory activities.

**Results:**

The EtOAc fraction markedly reduced the serum glucose level in the nicotinamide + STZ-induced type 2 diabetic rats (136.6 ± 12.17 mg/dL). Moreover, it showed a significant increase in serum insulin level (0.344 ± 0.04 µIU/mL). Four compounds (1—4) were isolated, of which luteolin showed potent inhibition of α-amylase activity, with an IC50 value of 19.32 ± 1.046 µM, as well as significant DPPH scavenging capacity. The LC-QTOF-MS/MS profiling validated the phytoconstituent composition and confirmed its chemotaxonomic relationship with closely related species.

**Conclusions:**

The antidiabetic effects of EtOAc fraction and its isolates may be attributed to its insulinotropic, antioxidant, and carbohydrate-hydrolysing enzyme inhibitory activities. Luteolin has been shown to be at least partially responsible for the antidiabetic activity of *B*. *tomentosa*.

**Supplementary Information:**

The online version contains supplementary material available at 10.1186/s12906-026-05484-2.

## Introduction

Diabetes mellitus is a chronic disease that occurs when the pancreas does not produce enough insulin, when the body cannot respond to the effects of insulin properly, or both. Type 2 diabetes (T2D) represents the most common form of diabetes. High blood sugar is a common effect of uncontrolled diabetes, and over time, it can cause severe damage to many of the body’s systems. The number of people with diabetes increased from 200 million in 1990 to 830 million in 2022. Prevalence has risen faster in low- and middle-income countries than in high-income countries. More than half of the patients aged 30 years and older who were living with diabetes were not using medication to treat their diabetes in 2022. Exploring natural products and traditional medicines represents a highly promising approach for drug discovery, as they serve as a vast reservoir of structurally diverse bioactive molecules capable of targeting complex metabolic pathways, given the massive global health burden of diabetes.

The genus *Bauhinia* L. (family Fabaceae) contains approximately 300 species distributed in tropical and subtropical regions [1, 2]. They are frequently used in Brazilian folk medicine to treat diabetes, inflammation, and pain [3, 4]. The members of the genus *Bauhinia* L. are small trees or medium to large shrubs. They are considered fast-growing plants that reach up to 4 m in height [5]. Leaves are light green, leathery, and bi-lobed, hence, they are known as cow’s hoof [5, 6]. *Bauhinia* L. is the most complex genus of the tribe Cercideae. Recently, it has been segregated into nine genera based on morphological, phylogenetic, and pollen studies [7–9].

*Bauhinia tomentosa* L. also named the yellow bell orchid tree, is a small tropical tree native to tropical and southern Africa. This species is widely distributed throughout sub-Saharan Africa and has been introduced and naturalized in several tropical and subtropical regions of Asia, including India and Sri Lanka [10]. In Egypt, *B. tomentosa* is cultivated as an ornamental plant in gardens, botanical collections, and research stations, rather than occurring as part of the native flora.

*B. tomentosa* plant is widely used in folk medicine in the treatment of diabetes, hyperlipidemia, diarrhea, fever, dysentery, large intestine problems, wounds, and snake bites [11, 12]. Previous phytochemical screening of different organs of *B. tomentosa* showed the presence of phenolic compounds, flavonoids, tannins, alkaloids, saponins, carbohydrates, fixed oils, proteins, amino acids, and mucilage [11, 12]. Several studies reported that the plant possesses different pharmacological activities such as antidiabetic, anti-inflammatory, antioxidant, antimicrobial, nephroprotective, and wound healing activities [12–15], which could be due to the high flavonoid and phenol contents [15–21].

While *B. tomentosa* has been utilized traditionally for metabolic disorders, a critical scientific gap remains in its systematic bioassay-guided fractionation, leaving its specific antidiabetic mechanisms largely uncharacterized. To address this, the current study coupled in vitro enzymatic assays with in vivo metabolic evaluations to elucidate the pathways through which the raw fractions and specific isolated compounds exert their glucose-regulatory effects. By pinpointing the active molecules within the most potent fraction, this work aims to transition *B. tomentosa* from a traditional empirical remedy to a molecularly validated source of novel antidiabetic leads.

## Materials and methods

### Phytochemical study

#### General experimental procedures

Spectra of 1D and 2D NMR were recorded using a Bruker AVANCE III 400 NMR spectrometer (Bruker, Karlsruhe, Germany). Separation of compounds was performed using CC packed with silica gel (60–120 mesh, Merck) and sephadex LH-20 (Mitsubishi Kagaku, Tokyo, Japan). Monitoring of fractions from CC was performed using preloaded TLC plates with silica gel 60 F_254_, 0.25 mm (Merck, Darmstadt, Germany). Detection was provided by using UV light (254 and 366 nm) followed by spraying with 1% vanillin - sulfuric acid and heating at 105 °C for 5–10 min [22].

#### Plant material

The aerial parts (leaves and stems) of *Bauhinia tomentosa* L. (Fabaceae) (https://www.worldfloraonline.org/taxon/wfo-0000213258) were collected in August 2019 from Mazhar’s Botanical Garden, Nahia, Imbaba, Giza, Egypt (30^o^ 02’ 34” N, 31^o^ 14’ 44” E). The plant material was authenticated by the Agricultural engineer, Therease Labib, Plant Taxonomy consultant at the Ministry of Agriculture. Permission for plant material collection was obtained from Shehab Mazhar Developments. The voucher specimens (AUN-PHG-102024) were deposited at The Herbarium of Pharmacognosy Department, Faculty of Pharmacy, Assiut University, Assiut, Egypt.

#### Extraction, fractionation, and isolation of constituents

The air-dried plant material of *B. tomentosa* (2 kg) was ground and macerated in 70% ethanol (7 × 3 L, 24 h each) at 25 ◦C. The combined ethanolic extracts were concentrated on a Büchi Rotavapor under reduced pressure at 45 °C to give a dry residue (50 g) which was dissolved in distilled water (1 L) and partitioned against dichloromethane (DCM) (5 × 1 L), then ethylacetate (EtOAc) (5 × 1 L) to yield two major fractions; DCM fraction 9 g (18%) and EtOAc fraction 4.5 g (9%). The remaining aqueous fraction was dried (Büchi Rotavapor under high vacuum, 30 g (60%).

Different plant fractions (DCM, EtOAc, and aqueous fractions) were subjected to preliminary in vivo evaluation of their antidiabetic activity. The EtOAc fraction was selected for further phytochemical investigation based on its superior antidiabetic activity in the in vivo study, particularly its marked reduction of serum glucose levels. Accordingly, it was subjected to chromatographic separation and compound isolation to identify the bioactive constituents responsible for the observed activity. The EtOAc fraction (4.5 g) was chromatographed over Sephadex LH-20 (100 × 2.7 cm, 90 g) using methanol (MeOH) [2 L] to yield nine subfractions I-IX. Subfraction III (1.2 g) was applied on silica gel CC (120 × 1.7 cm, 30 g) and eluted with DCM-MeOH [10:0 (1 L), 9.5:0.5 (1 L), 9:1 (1 L), 8.5:1.5 (1 L), and 8:2 (1 L)], yielding compound 1 (80 mg). Subfraction IV (1.1 g) was chromatographed over silica gel CC (120 × 1.7 cm, 30 g) and eluted with DCM-MeOH [10:0 (1 L), 9.5:0.5 (1 L), 9:1 (1 L), 8.5:1.5 (1 L), and 8:2 (1 L)], eluting compound 2 (7.7 mg). Subfraction V (0.5 g) was purified over silica gel CC (80 × 1.5 cm, 15 g) and eluted with DCM-MeOH [10:0 (1 L), 9.5:0.5 (1 L), and 9:1 (1 L)], eluting compound 3 (6.7 mg). Subfraction VIII (0.7 g) was chromatographed over silica gel CC (80 × 1.5 cm, 20 g) and eluted with DCM-MeOH [10:0 (1 L), 9.5:0.5 (1 L), and 9:1 (1 L)], eluting compound 4 (27 mg), see Scheme S1.

#### LC-QTOF-MS/MS system

Chromatographic separation was conducted via an Inertsil ODS-3 (100 mm × 2.1 mm, 3 μm) column (GL-Science, Torrance, CA, USA) on an Exion LC (Shimadzu, Quebec, Canada) with a flow rate of 0.35 mL/min. The column temperature was 50 °C. The mobile phase consisted of solvent A (0.1% formic acid in water) and solvent B (acetonitrile) for a total run time of 50 min. The analysis was carried out by applying the following binary gradient at a flow rate of 0.35 mL/min: 0–5 min, isocratic 5% B (acetonitrile), 95% A (water/formic acid, 99.9/0.1 [v/v]), 5–25 min, linear from 5% to 95% B, 25–30 min isocratic 95% B, 31–32 linear from 95% to 5% B; 33–40 min, isocratic 5% B. The mass spectrometry (MS) analysis was performed using a Sciex X500 QTOF (AB Sciex LLC, Marlborough, U.S.A.) in the electron spray (ESI) negative mode; capillary voltage of 4000 V; nebulizer pressure: 2.0 bar; drying gas: 8 L/min at 300 °C.

#### Data collection and processing

The data acquisition and analysis were performed via SCIEX OS2.0.1 software. Peak picking was performed via the auto algorithm, which is the optimal option for metabolomics studies. Chromatograms within the batch being processed are evaluated to determine which sample provides the best peak model MS/MS library for each transition. The peak model is constructed from a 3 Gaussian peak model. Metabolite annotation was performed according to the confidence levels proposed by Schymanski et al. (2014). The metabolites reported in this study were assigned to Level 2 on the basis of high-resolution accurate mass, isotope distributions, MS/MS fragmentation spectra, and comparisons with the library spectra via the SCIEX All-in-One HRMS/MS library version 2.0, SCIEX Accurate Mass Metabolite Spectral Library version 2.0, and NIST 2017 [23]. We did not consider the retention time (RT) as the primary parameter because it varies with the liquid chromatography setup. Additionally, the matrix effect of the sample may shift the RTs of the compounds when we inject the entire fraction without any pretreatment to achieve optimal untargeted metabolomics screening.

### Evaluation of antidiabetic and antioxidant activity

#### Materials

Streptozotocin was purchased from MP Biomedicals (California, USA). Nicotinamide was purchased from ACROS Organics (Belgium). A rat insulin ELISA kit was purchased from YL Biont (Shanghai YL Biont Co., Ltd., China; Cat. No. YLA0037RA). A rat GLUT4 ELISA kit was purchased from YL Biont (Shanghai YL Biont Co., Ltd., China, Cat. No. YLA0671RA). A serum glucose detection kit was purchased from Spectrum (Cairo, Egypt). The enzyme *α*-glucosidase (from *Saccharomyces cerevisiae*), the substrate para-nitrophenyl *β*-ᴅ-glucopyranoside, the *α*-amylase enzyme (from porcine pancreas), the substrate 2-chloro-4-nitrophenyl-*α*-ᴅ-maltotrioside, and DPPH (2,2-diphenyl-1-picryl-hydrazyl-hydrate) were all purchased from Sigma‒Aldrich.

#### Animals

Male Wistar rats (average weight 150–200 g) were used for the induction of T2D. Animals were purchased from the Assiut University animal care facility and kept there until euthanasia. They were acclimatized to controlled room temperature (25 °C) and humidity (65–75%) under a 12 h:12 h light–dark cycle, and tap water and diet were freely accessed *ad libitum*. All experiments were conducted in compliance with protocols approved by the Institutional Animal Care and Use Committee guidelines (Ethics committee, Faculty of Pharmacy, Assiut University; 05-2024-001).

### In vivo study and biochemical estimations

#### Induction of type 2 diabetes (T2D)

Diabetes was induced in overnight-fasted rats by a single IP injection of 110 mg/kg of nicotinamide in normal saline, and 15 min later by IP injection of 65 mg/kg of STZ dissolved in freshly prepared 0.1 M citrate buffer, with a pH of 4.4 [24]. The rats received 10% w/v sucrose solution in their drinking water for the first 24 h to avoid hypoglycemia. Seventy-two hours following nicotinamide + STZ injections, blood glucose levels from overnight-fasted rats were measured via single tail tip pricksprick using glucose test strips and a glucometer (Smart Test, Taiwan). Rats with fasting blood glucose levels ≥ 250 mg/dL were deemed diabetic and used for further experiments.

#### Animal groups

Animals were randomly divided into five groups; each group consisted of 6 rats. Group I was injected once IP with 0.1 mL of 0.1 M citrate buffer (pH 4.4) and served as a normal control (NC+vehicle). Following the confirmation of hyperglycemia, the T2D rats were randomly divided into 4 groups of 6 rats each. The rats in group II, which were injected with nicotinamide + STZ, received Tween 80 (1% v/v) *p.o.* once daily and served as the T2D control (T2D+vehicle). Rats of group III, IV, and V, which received nicotinamide + STZ injections, were administered the aqueous, EtOAc, and DCM fractions of *B. tomentosa*, respectively. The fractions were given orally by gavage, as an emulsified form in 1% Tween 80, at doses of 300 mg/kg. The dose (300 mg/kg/day) was selected on the basis of previous studies, which demonstrated significant antidiabetic activity of the aqueous extract of *Bauhinia tomentosa*, as well as the different extracts of *Bauhinia* species in rodent models at this dose [25–27]. All the groups received the tested drugs once daily for the remaining 14 days of the experimental period.

#### Specimen collection

At the end of the 14-day treatment period, the animals were euthanized via decapitation under ketamine anaesthesia, along with transcardial perfusion with cold phosphate-buffered saline (PBS; pH 7.4). Blood samples were withdrawn for separation of serum and then stored at -20 °C until further use. Skeletal muscle samples (gastrocnemius muscle) were isolated from each animal, rinsed well in PBS, blotted dry on filter paper, weighed, and stored at -80 °C for further biochemical measurements. Upon testing, the tissues (50 mg each) were homogenized in PBS with an electric homogenizer and then centrifuged at 10,000 rpm for 10 min at 4 °C to remove the cellular debris. The supernatants were then snap-frozen in liquid nitrogen and stored at -20 °C.

#### Serum analysis of glucose and insulin

Determination of serum glucose and insulin levels was conducted. Serum glucose levels were measured by commercially available glucose oxidase assay kit. Serum insulin levels were determined via a rat insulin enzyme-linked immunosorbent assay (ELISA) kit in accordance with the manufacturer’s instructions. The assay is based on a sandwich ELISA format with HRP-mediated colorimetric detection, and optical density readings were used to calculate insulin concentrations from a standard curve.

#### Estimation of skeletal muscle GLUT4 levels

GLUT4 protein levels in muscle homogenates were quantified via an ELISA kit according to the manufacturer’s protocol. This kit uses an ELISA based on Biotin double-antibody sandwich technology. Briefly, samples were added to wells, which are pre-coated with GLUT4 monoclonal antibody and then incubated. After that, a biotin-labelled anti-GLUT4 detection antibody was added, followed by streptavidin-HRP, which binds the biotin and forms an immune complex. Unbound enzymes were removed after incubation and washing. The substrates were then added, resulting in a color change. The intensity of the color, measured by optical density, is proportional to the concentration of GLUT4 in the samples.

### In vitro study of isolated compounds

#### Antioxidant activity using 2, 2-diphenyl-1-picrylhydrazyl (DPPH)

The DPPH scavenging capacity of the isolated compounds was quantified following the reported method of Elkholy et al. [28]. 100 µL of freshly prepared DPPH solution (0.1% in MeOH) was added to 100 µL of different concentrations of the samples and incubated at room temperature for 30 min in the dark. MeOH was used as a blank, while Trolox was used as a positive control. The assay relies mainly on the disappearance of the purple colour of DPPH radicals when they are reduced. The absorbance was measured at 540 nm, and the results were recorded using a microplate reader (FluoStar Omega, Germany).

The reduction in DPPH color intensity was represented via the following equation:

$$\begin{aligned}\%\mathrm{Inhibition}=&\left[\left(\text{Average absorbance of blank}-\text{average absorbance}\:\text{of the sample}\right)\right.\\&\left./\left(\text{Average absorbance of the blank}\right)\right]\times100.\end{aligned}$$

#### In vitro inhibition assay of α-glucosidase activity

The modified approach of Lee et al. was utilized to measure the inhibitory activity of the *α*-glucosidase enzyme. The reaction was carried out at 37 °C by incubating 25 µL of the sample mixture with 50 µL of α-glucosidase from *Saccharomyces cerevisiae* (0.6 U/mL) and 25 µL of 3 mM *p*-nitrophenyl-*α*- ᴅ-glucopyranoside as the substrate. After 30 min, 0.1 M Na_2_CO_3_ solution was added to stop the reaction, and the enzymatic activity was determined by measuring the absorbance at a wavelength of 405 nm via a microplate reader (FluoStar Omega, Germany). Acarbose was used as a positive control, and all the outcomes were expressed as IC_50_ values [29].

#### In vitro inhibition assay for α-amylase activity

In accordance with the method of Khadayat and coworkers [30], an *α*-amylase in vitro inhibitory assay was carried out. Briefly, samples/blank were mixed with 140 µL of phosphate buffer (pH 7). To this mixture, 20 µL of *α*-amylase from porcine pancreas (Sigma‒Aldrich, A3176) prepared at 1 mg/mL (manufacturer-specific activity: ≥5 U/mg solid) in the same buffer was added, and the mixture was incubated for 15 min at 37 °C. Then, 20 µL of substrate in phosphate buffer was added, and the mixture was incubated again for 10 min at 37 °C. Enzyme activity was determined by measuring the release of *p*-nitroanilline from the substrate at 405 nm via a microplate reader (FluoStar Omega, Germany). The percentage of inhibition of *α*-amylase was calculated according to the following equation:

$$\%\mathrm{Inhibition}=\left[\left(\text{A blank}-\text{A sample}\right)/\text{A blank}\right]\times100$$

where A blank is the absorbance of the control (blank, without inhibitor) and A sample is the absorbance in the presence of the inhibitor.

### Statistical analysis

The data are expressed as the mean ± standard error (SE). Statistical analyses for the in vivo parameters were conducted by using a one-way analysis of variance (ANOVA), followed by Tukey’s multiple-comparisons *post hoc* test, in GraphPad Prism version 5.03 (GraphPad Software, Inc.). Differences were considered statistically significant at a *P* value < 0.05 for all comparisons [31]. For in vitro enzymatic and antioxidant assays, IC_50_ values were estimated using non-linear regression analysis of concentration‒response curves in GraphPad Prism. Differences among the fitted curves were evaluated using the extra sum-of-squares F test (global nonlinear regression curve comparison). Pairwise curve comparisons were performed where appropriate, and *p* < 0.05 was considered statistically significant.

## Results

### Phytochemical investigation

**Fig. 1:**
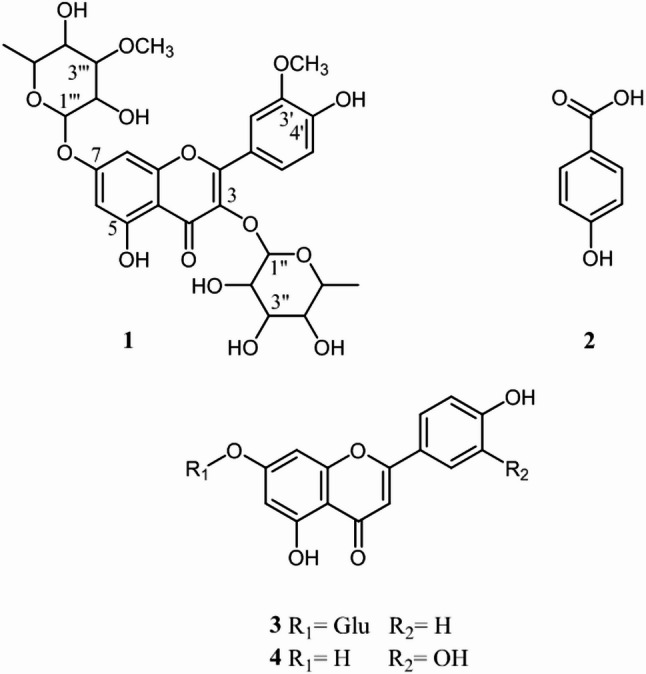
Chemical structures of the isolated metabolites (1─4)

The antidiabetic-guided fractionation of *B. tomentosa* aerial parts alcoholic extract was performed, and the results of the serum analysis and skeletal muscle GLUT4 level were used to select the bioactive fraction to characterize and isolate its constituents. The EtOAc fraction has the most potent antidiabetic activity, as it markedly reduced the serum glucose levels in the nicotinamide + STZ-induced T2D rats (136.6 ± 12.17 compared with 496.0 ± 36.6 in the T2D rats, *p <* 0.01). Thus, it was analysed by using LC-QTOF-MS/MS and fractionated by using different chromatographic techniques yielding isorhamnetin-3-*O*-*α*-ʟ-rhamnoside-7-*O*- *α-*ʟ-3-methyl rhamnoside (1), *p*-hydroxybenzoic acid (2), apigenin-7-*O*-*β*-ᴅ-glucopyranoside (3), and luteolin (4) (Fig. 1).

The ^1^H-NMR and ^13^C-NMR data of the isolated compounds are presented in Table 1; Figs. 2, S1-S11.

**Fig. 2:**
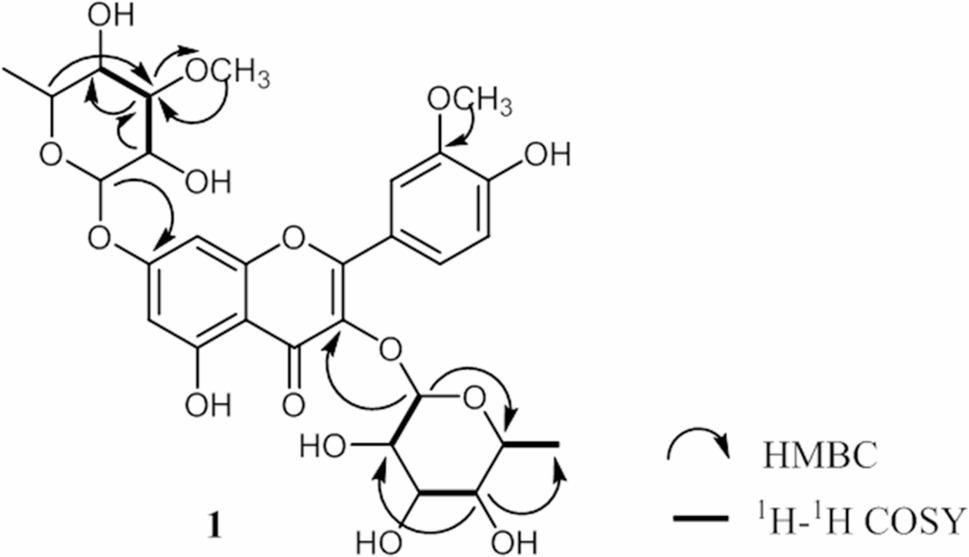
Key HMBC and ^1^H–^1^H COSY correlations of compound 1

**Table 1:** NMR spectroscopic data of compound 1 (DMSO-*d*_*6*_, 400 MHz for ^1^H, 100 MHz for DEPT Q 135, *δ* in ppm)

Position	δ_C_	δ_H_ (J in Hz)	Position	δ_C_	δ_H_ (J in Hz)
2	157.9		1′′	102.0	5.29 (d, 1.6)
3	134.7		2′′	70.2	3.98 (d, 2.9)
4	178.1		3′′	72.5	3.64 (d, 2.8)
5	161.1		4′′	72.1	3.31 (d, 8.5)
6	99.6	6.46 (d, 2.1)	5′′	71.1	3.16 (m)
7	161.9		6′′	17.6	0.80 (d, 5.0)
8	94.9	6.81 (d, 2.1)	1′′′	98.5	5.56 (d, 1.8)
9	156.3		2′′′	72.7	3.36 (d, 9.3)
10	105.9		3′′′	83.9	3.01 (t, 9.3)
1′	120.7		4′′′	70.2	3.51 (d, 8.6)
2′	112.8	7.46 (d, 2.1)	5′′′	71.1	3.64 (m)
3′	147.5		6′′′	18.1	1.13 (d, 6.1)
4′	149.9		3′′′ OCH_3_	59.8	3.44 (s)
5′	115.6	6.94 (d, 8.0)			
6′	122.9	7.43 (dd, 2.1, 8.2)			
3′ OCH_3_	55.9	3.85 (s)			

Compound 1 was obtained as a yellowish residue (MeOH), with UV (MeOH) λ_max_ (log *ε*) values of 265 (1.53) and 346 (1.49) nm and [α]^20^_D_ -175 (c 0.2, MeOH). IR analysis (KBr disc) revealed peaks at 3413 cm^− 1^ (–OH phenolic and sugar hydroxyl groups), 1652 (C = O carbonyl), 1607 (C = C aromatic), 1397 (CH_3_), 1209 (C-O), and 1063 (C-O-C) (Fig. 7S). The ^1^H-NMR and DEPT Q 135 spectra of compound 1 (Table 1, Supplementary data) revealed the characteristic signals of the isorhamnetin nucleus [32, 33], along with two sugar units. One of the sugar moieties was identified as *α*-ʟ-rhamnoside from the characteristic signals of its anomeric carbon at *δ*_C_ 102.0 along with its corresponding proton at *δ*_H_ 5.29 (d, *J* = 1.6 Hz) and the methyl group at *δ*_H_ 0.80 (d, *J* = 5.0 Hz)/*δ*_C_ 17.6. The rhamnose moiety attached to the isorhamnetin nucleus was assigned to be at C-3 on the basis of the HMBC correlation between H-1′′ and C-3 (*δ*_C_ 134.7) (Fig. 2). The second sugar unit contained a methoxy group (*δ*_H_ 3.44 and *δ*_C_ 59.8) attached to C-3′′′ of another *α*-ʟ-rhamnoside unit, which was identified as 3-methyl rhamnose [34]. The linkage of the methoxy group was established through the HMBC correlation between H-3′′′ (*δ*_H_ 3.01 t, *J* = 9.3 Hz) and the methoxy carbon (*δ*_C_ 59.8), as well as by the downfield shift of C-3′′′ (*δ*_C_ 83.9). The ^1^H-^1^H COSY spectrum further revealed proton assignments for the methyl rhamnose moiety, showing correlations from H-2′′′ (*δ*_H_ 3.36, d, *J* = 9.3 Hz) to H-3′′′ (*δ*_H_ 3.01, t, *J* = 9.3 Hz) and from H-3′′′ to H-4′′′ (*δ*_H_ 3.51, d, *J* = 8.6 Hz) (Fig. 2). The HMBC spectrum also confirmed the attachment of the 3-methyl rhamnose to C-7 of the isorhamnetin nucleus, based on the HMBC correlation between the anomeric proton H-1′′′ (*δ*_H_ 5.56, d, *J* = 1.8 Hz) and C-7 (*δ*_C_ 161.9).

**Fig. 3:**
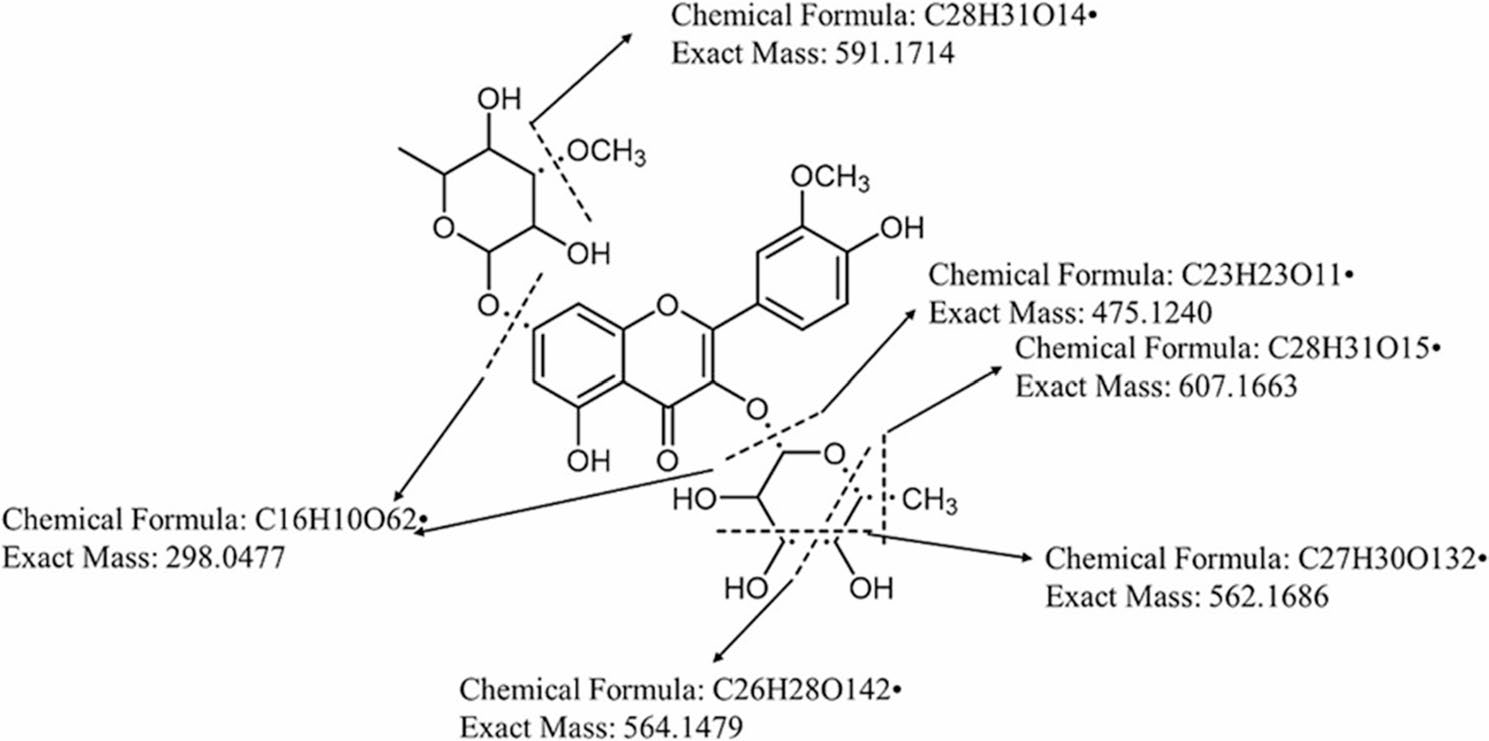
Proposed fragmentation pattern of compound 1

The previous findings were further confirmed by LC-QTOF-MS/MS in negative mode. Compound 1 was recorded at R_t_ 12.91 min and produced a deprotonated molecular ion at m/z 621.2256. On the basis of the presence of a characteristic fragment at m/z 299 in the MS/MS spectrum, as well as a major fragment at m/z 269 corresponding to [M-CH_3_-CO], the aglycone was tentatively identified as the methoxylated flavonoid isorhamnetin. The presence of a methyl rhamnose unit was confirmed by the fragment at m/z 475, representing isorhamnetin with a methyl rhamnose moiety [35]. Additionally, fragments at m/z 562 and 591 supported the presence of a second rhamnose unit. These findings, as illustrated in Fig. 3, suggested that the compound is a glycoside of isorhamnetin with two rhamnose moieties and a methyl group.

Based on this spectral data, compound 1 was identified as isorhamnetin-3-*O*-*α*-ʟ-rhamnoside-7-*O*-*α-*ʟ-3-methyl rhamnoside.

Three known compounds have been isolated and identified as *p*-hydroxybenzoic acid 2 [36], apigenin-7-*O*-*β*-ᴅ-glucopyranoside 3 [37], and luteolin 4 [38] based on their spectroscopic data analysis (Supplementary data) and by comparing these data with literature values. The presence of characteristic ions (m/z) in the LC‒QTOF‒MS/MS spectrum confirmed the presence of the isolated compounds.

### Characterization of Bauhinia metabolites via LC‒QTOF‒MS/MS

LC-QTOF-MS/MS analysis of the EtOAc extract from the *Bauhinia* sample revealed the presence of 68 compounds. The compounds were putatively annotated by comparing the detected compounds’ MS data (accurate mass and fragmentation in negative ion mode), as well as collision energy, with the library (SCIEX All-in-One HRMS/MS library version 2.0). They were all at confidence level 2 according to Schymanski’s identification confidence levels [23]. Furthermore, the characterized compounds, or their structurally related derivatives, have previously been reported from *Bauhinia* species or other members of the family, supporting their chemotaxonomic significance for the genus and the family. The isolated compounds were successfully characterized and are summarized in Table 2 according to the applied classification criteria.

**Table 2:** Putatively identified metabolites in the EtOAc fraction of *Bauhinia tomentosa* aerial parts using LC-QTOF-MS/MS in negative ionization mode. All compounds were annotated at a level 2 confidence level according to Schymanski’s identification confidence levels

Compound Name	Ionized Ions	RT (Minutes)	Deprotonated molecular formula	Calculated Mass (Dalton)	Measured Mass (M) (Dalton)	Error m/z (Δppm)
Phenolic compounds						
(-)-Quinic acid	[M-H]^−^	2.84	C_7_H_12_O_6_	192.0898	191.0825	-2.81
Oxyresveratrol	[M-H]^−^	4.51	C_14_H_12_O_4_	244.1023	243.0951	-2.58
Xanthotoxol	[M-H]^−^	5.68	C_11_H_6_O_4_	202.0074	200.9999	-1.58
*p*-Hydroxybenzoic acid	[M + Cl]^−^	6.14	C_7_H_6_O_3_	138.03169	173.0011	0
*N*-Caffeoyl-*O*-methyltyramine	[M-H]^−^	6.51	C_18_H_19_NO_4_	313.1441	312.1367	-1.25
Arbutin	[M-H_2_O-H]^−^	6.63	C_12_H_16_O_7_	290.1365	271.1187	-2.05
1,2,3-Benzenetriol pyrogallol	[M-H]^−^	9.28	C_6_H_6_O_3_	126.05	125.043	-6.27
Gallic acid	[M-H]^−^	9.39	C_7_H_6_O_5_	170.0459	169.0383	-1.12
3-*O*-Methylgallic acid	[M-H]^−^	10.71	C_8_H_8_O_5_	184.0633	183.0556	-0.81
Salidroside	[M-H]^−^	10.87	C_14_H_20_O_7_	300.1245	299.1165	0.50
4’,6’-Dimethoxy-2’-hydroxyacetophenone (Xanthoxylin)	[M-H]^−^	11.21	C_10_H_12_O_4_	196.0647	195.0569	-0.15
Pyrocatechol	[M]^−^	11.24	C_6_H_6_O_2_	109.0455	109.0455	0
Coniferyl aldehyde	[M-H]^−^	11.43	C_10_H_10_O_3_	178.0884	177.0805	0.28
4-Hydroxyphenylpyruvic acid	[M-H]^−^	11.47	C_9_H_8_O_4_	180.0889	179.0811	0.17
*p*-Hydroxyphenyl lactic acid	[M-H]^−^	11.47	C_9_H_10_O_4_	182.0833	181.0763	-4.23
4-Methylcatechol	[M-H]^−^	12.04	C_7_H_8_O_2_	124.0708	123.0629	0.40
Homogentisic acid	[M-H]^−^	12.04	C_8_H_8_O_4_	168.0661	167.0585	-1.37
3,4-Dihydroxybenzaldehyde (Protocatechualdehyde)	[M-H]^−^	12.15	C_7_H_6_O_3_	138.0881	137.0795	5.65
Rosmarinic acid	[M + Cl]^−^	12.42	C_18_H_16_O_8_	324.1754	359.1445	-0.76
3-Methoxycinnamic acid	[M-H]^−^	13.21	C_10_H_10_O_3_	178.0516	177.0441	-1.57
5-Hydroxyisovanillic acid	[M-H]^−^	13.21	C_8_H_8_O_5_	184.1351	183.128	-3.91
Phloretin	[M-H]^−^	13.25	C_15_H_14_O_5_	274.1204	273.1131	-1.93
2-Methoxy-5-methylphenol	[M-H]^−^	13.55	C_8_H_10_O_2_	120.0752	119.0676	-1.58
5-2-(3,5-dimethoxyphenyl) ethenyl-2-methoxy-phenol	[M-H]^−^	14.08	C_17_H_18_O_4_	286.2156	285.2074	1.50
Resveratrol-3-*O*-sulfate	[M-H]^−^	14.84	C_14_H_12_O_6_S	308.1708	307.1645	-4.80
Flavonoids						
Herbacetin (8-Hydroxykaempferol)	[M-H]^−^	2.2	C_15_H_10_O_7_	301.8967	300.8915	-8.61
2,4-Dimethoxy-2’-hydroxychalcone	[M-H]^−^	2.27	C_17_H_16_O_4_	283.9535	282.9452	1.87
3’,4’-Dimethoxy-3-hydroxy-6-methylflavone	[M-H]^−^	10.07	C_18_H_16_O_5_	312.1825	311.1753	-1.86
Scutellarein (6-Hydroxyapigenin)	[M-H]^−^	11.43	C_15_H_10_O_6_	286.1433	285.1359	-1.26
Robinin	[M-H]^−^	12.11	C_33_H_40_O_19_	740.3085	739.3009	-0.28
Neohesperidin	[M-H]^−^	12.42	C_28_H_34_O_15_	610.2283	609.2196	1.39
Kaempferitrin	[M-H]^−^	12.61	C_27_H_30_O_14_	578.2318	577.2242	-0.42
Isoquercitin	[M-H]^−^	12.76	C_21_H_20_O_12_	464.1548	463.1469	0.17
Cynaroside (Luteolin 7-glucoside)	[M-H]^−^	12.76	C_21_H_20_O_11_	448.156	447.1484	-0.49
Isorhamnetin 3-neohesperidoside	[M-H_2_O-H]^−^	12.8	C_28_H_32_O_16_	642.2573	623.239	-0.15
Apiin	[M-H]^−^	12.91	C_26_H_28_O_14_	564.2176	563.2136	-6.79
Nepetin 7-glucoside	[M-H]^−^	13.17	C_22_H_22_O_12_	478.1725	477.1649	-0.46
Frangulin A	[M-H]^−^	13.21	C_21_H_20_O_9_	416.2584	415.2509	-0.62
Tectoridin	[M-H]^−^	13.33	C_22_H_22_O_11_	462.1752	461.1676	-0.50
Apigenin 7-glucoside	[M-H]^−^	13.51	C_21_H_20_O_10_	432.1255	431.1177	0.16
Taxifolin	[M-H]^−^	13.55	C_15_H_12_O_7_	304.0988	303.0909	0.16
Quercitrin	[M-H]^−^	13.67	C_21_H_20_O_11_	448.1589	447.1508	0.74
Tricetin	[M-H]^−^	14.04	C_15_H_10_O_7_	302.0832	301.0754	0.13
4-Benzyloxy-2’-hydroxy-3,4’,6’-trimethoxychalcone	[M-H]^−^	14.27	C_25_H_24_O_6_	420.1753	419.1676	-0.29
Eriodictyol	[M-H]^−^	14.88	C_15_H_12_O_6_	288.1	287.0916	1.91
Kaempferol	[2 M-H]^−^	14.92	C_15_H_10_O_6_	286.0862	285.0789	-1.78
Luteolin	[M-H]^−^	14.95	C_15_H_10_O_6_	286.0849	285.0773	-0.66
Quercetin	[M-H]^−^	14.99	C_15_H_10_O_7_	302.0828	301.0751	-0.26
Isorhamentin	[M-H]^−^	15.26	C_16_H_14_O_7_	316.1003	315.0926	-0.19
Quercetin 3,4’-dimethyl ether	[M-H]^−^	15.45	C_17_H_14_O_7_	330.1176	329.1097	0.15
Genkwanin (7-*O*-Methylapigenin)	[M-H]^−^	17.98	C_16_H_12_O_5_	284.1068	283.1008	-6.37
Wogonin	[M-H]^−^	21.27	C_16_H_12_O_5_	284.2154	283.208	-1.55
Miscellaneous						
Galactonic acid	[M-H]^−^	2.54	C_6_H_12_O_7_	196.0857	195.0782	-1.48
Dulcitol (galactitol)	[M-H]^−^	2.54	C_6_H_14_O_6_	182.1043	181.0966	-0.44
Threonic acid	[M-H]^−^	2.73	C_4_H_8_O_5_	136.0572	135.0493	0.44
DL-Trehalose	[M-H]^−^	2.77	C_12_H_22_O_11_	342.1597	341.1533	-3.97
DL-Ribose	[M-H]^−^	2.92	C_5_H_10_O_5_	150.0743	149.0666	-0.73
Glucaric acid	[M-H]^−^	3.03	C_6_H_10_O_8_	210.1029	209.0953	-0.81
Oxypurinol	[M-H]^−^	5.26	C_5_H_4_N_4_O_2_	152.0551	151.0479	-3.88
DL-Malic acid	[M-H]^−^	6.36	C_4_H_6_O_5_	134.0773	133.07	-3.43
L-Phenylalanine	[M-H]^−^	10.11	C_9_H_11_NO_2_	165.1025	164.0947	-0.12
4-Methyl-2-oxovaleric acid	[M-H]^−^	10.34	C_6_H_10_O_3_	130.0459	129.038	0.46
Pantothenic acid	[M-H]^−^	10.53	C_9_H_17_NO_5_	219.141	218.1334	-1.05
Glutaric acid	[M-H]^−^	10.56	C_5_H_8_O_4_	132.0612	131.0539	-3.63
2-Hydroxy-2-methylbutyric acid	[M-H]^−^	11.36	C_5_H_10_O_3_	118.0804	117.0731	-4.49
1-Oleoyl-L-lysophosphatidic acid	[M-H]^−^	12.19	C_21_H_41_O_7_P	436.2862	435.2784	-0.09
Fatty Acids						
Heptadecanoic acid	[M-H]^−^	13.86	C_17_H_34_O_2_	270.0895	269.0819	-0.63
3-Hydroxyoctadecanoic acid	[M-H]^−^	16.85	C_18_H_36_O_3_	300.1034	299.0955	0.20

All the compounds have a mass error of less than 10 ppm. The compound name, retention time (RT) in minutes, calculated mass and measured mass (M) in Daltons, and mass error (Δ ppm) are listed. Additionally, the molecular formula and ionization ions of each compound are listed in Table 2.

### In vivo and in vitro antidiabetic activity

#### Effect of treatment with different B. tomentosa fractions on fasting serum glucose and insulin levels in T2D rats

**Fig. 4:**
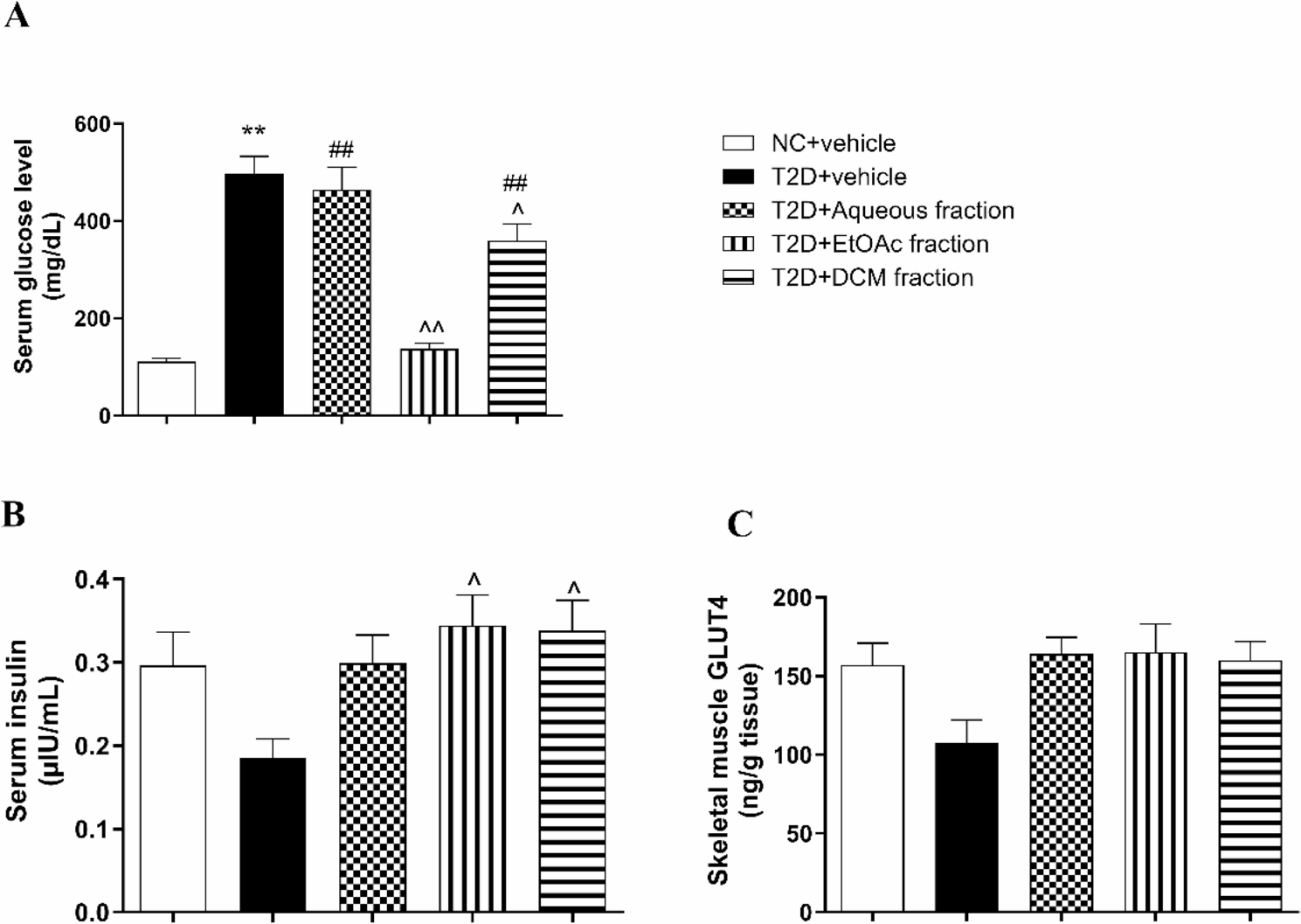
Impact of treatment with *B. tomentosa* fractions on serum glucose (**A**), insulin (**B**) and skeletal muscle GLUT4 (**C**) levels in T2D rats. NC: Normal Control. T2D: type 2 diabetes. EtOAc: Ethyl acetate. DCM: Dichloromethane. Values are represented as mean ± SE for *n* = 6. ^**^p < 0.01 vs. NC; ^^^^*p* < 0.01, ^^^*p* < 0.05 vs. T2D + vehicle and ^##^*p* < 0.01 vs. T2D + EtOAc fraction

Nicotinamide + STZ injection resulted in stable hyperglycemia, with the T2D group showing significantly higher serum glucose levels (496.0 ± 36.6 mg/dL) compared to the normal control group (110.6 ± 7.72 mg/dL, *p* < 0.0001; Table 3; Fig. 4A). While the T2D group also displayed a trend toward hypoinsulinemia (0.185 ± 0.02 µIU/mL vs. 0.296 ± 0.03 µIU/mL), this difference was not statistically significant (*p* = 0.1779, Table 4; Fig. 4B).

**Table 3:** Reduction in fasting serum glucose levels by different *B. tomentosa* fractions in T2D rats

Treated group	Serum glucose level (mg/dL)
NC+vehicle	110.6 ± 7.72
T2D+vehicle	496.0 ± 36.6**
T2D+Aqueous fraction	463.8 ± 46.94^##^
T2D+ EtOAc fraction	136.6 ± 12.17^^
T2D + DCM fraction	359.2 ± 34.00^ ^##^

*NC* Normal Control, *T2D* type 2 diabetes, *EtOAc* Ethyl acetate, *DCM* Dichloromethane. Data are represented as mean ± SE (*n* = 6)

^**^p < 0.01 vs. NC, ^^^^*p* < 0.01, ^^^*p* < 0.05 vs. T2D + vehicle and ^##^*p* < 0.01 vs. T2D + EtOAc fraction (ANOVA)

The current study revealed that treatment with different fractions of *B. tomentosa* effectively reduced serum glucose levels in nicotinamide + STZ-induced T2D rats. The effect was most marked with the EtOAc fraction (136.6 ± 12.17 compared with 496.0 ± 36.6 in T2D, *p <* 0.0001). A significant but less pronounced effect was observed with the DCM fraction, which reduced glucose levels to 359.2 ± 34.00 mg/dL (*p* = 0.0358; *p* < 0.05). In contrast, the aqueous fraction showed no significant reduction, with the serum glucose level remaining at 463.8 ± 46.94 mg/dL (*p* = 0.9484; Table 3; Fig. 4A).

Regarding serum insulin, levels were significantly increased with the EtOAc and DCM fractions (0.344 ± 0.04 and 0.338 ± 0.04, respectively; *p <* 0.05), when compared to untreated T2D group (*p* = 0.0224 and *p* = 0.0301, respectively) (Table 4; Fig. 4B).

**Table 4:** Effect of treatment with different *B. tomentosa* fractions on serum insulin levels in diabetic rats

Treated group	Serum insulin (µIU/mL)
NC+vehicle	0.296 ± 0.03
T2D+vehicle	0.185 ± 0.02
T2D+Aqueous fraction	0.299 ± 0.03
T2D+EtOAc fraction	0.344 ± 0.04^
T2D + DCM fraction	0.338 ± 0.04^

*NC* Normal Control, *T2D* type 2 diabetes, *EtOAc* Ethyl acetate, *DCM* Dichloromethane. Data are represented as mean ± SE (*n* = 6)

^ *p <* 0.05 compared to T2D (ANOVA)

Comparison of the different *B. tomentosa* fractions revealed that the EtOAc fraction presented significantly greater glucose-lowering activity compared to the other *B. tomentosa* fractions (*p* < 0.0001 vs. the aqueous fraction and *p* = 0.0003; *p* < 0.01 vs. DCM). However, the serum insulin levels did not differ significantly between the EtOAc fraction and the other fractions (*p* = 0.8789 vs. the aqueous fraction and *p* > 0.9999 vs. the DCM; *p* > 0.05). Additionally, no statistically significant differences existed between the effects of the aqueous and DCM fractions with respect to any of the measured parameters (*p =* 0.1590 for serum glucose and 0.9250 for serum insulin; *p* > 0.05).

#### Impact of treatment with B. tomentosa fractions on skeletal muscle GLUT4 levels in T2D rats

The results revealed a slight nonsignificant reduction in GLUT4 levels in the skeletal muscle tissue homogenates of the nicotinamide + STZ-injected rats compared with those of the normal control rats (107.8 ± 14.36 in the T2D group vs. 157.0 ± 14.02 ng/g tissue in the NC group; *p* = 0.1259). Additionally, a modest increase in these levels was detected following treatment with different fractions of *B. tomentosa* (165.0 ± 17.96, 164.0 ± 10.56 and 159.8 ± 11.92 ng/g tissue for the EtOAc, aqueous, and DCM fractions, respectively), but this increase was non-significant, compared with that in diabetic animals (*p* = 0.0551, 0.0616 and 0.0947, respectively). Similarly, GLUT4 levels did not show a significant difference either between the EtOAc fraction and the other fractions (*p* > 0.9999 vs. the aqueous fraction and *p* = 0.9989 vs. DCM; *p* > 0.05) or between the aqueous and DCM fractions (*p* = 0.9995, Table 5; Fig. 4C).

**Table 5:** Skeletal muscle GLUT4 levels in diabetic rats following treatment with *B. tomentosa* fractions

Treated group	Skeletal muscle GLUT4 (ng/g tissue)
NC+vehicle	157.0 ± 14.02
T2D+vehicle	107.8 ± 14.36
T2D+Aqueous fraction	164.0 ± 10.56
T2D+ EtOAc fraction	165.0 ± 17.96
T2D + DCM fraction	159.8 ± 11.92

*NC* Normal Control, *T2D* type 2 diabetes, *GLUT4* glucose transporter 4, *EtOAc* Ethyl acetate, *DCM* Dichloromethane. Data are represented as mean ± SE (*n* = 6)

#### In vitro antioxidant, α-glucosidase, and α-amylase inhibitory activities of the isolated B. tomentosa compounds

In our study, the isolated compounds exhibited significant in vitro antioxidant activity, with compound 4 being the most potent, followed by compound 2 (*p* < 0.0001 vs. compounds 1, 2 and 3; Table 6).

**Table 6:** Estimated IC_50_ values of the antioxidant, *α*-glucosidase and *α*-amylase inhibitory activities of the isolated *B. tomentosa* compounds

Sample	Antioxidant activity		α-glucosidase inhibitory activity		α-amylase inhibitory activity	
	IC_50_^†^	SE	IC_50_^†^	SE	IC_50_^†^	SE
Compound 1	789.6**^##^	1.048	NA	NA	288.3**^##^	1.039
Compound 2	329.7**^##^	1.082	NA	NA	NA	NA
Compound 3	784.0**^##^	1.049	NA	NA	41.5**^##^	1.056
Compound 4	135.1**	1.029	708.3**	1.025	19.32**	1.046
Positive control	26.43	1.029	141.7	1.033	6.47	1.034

^†^ IC_50_ values are expressed in µM and were estimated from nonlinear regression analysis of concentration–response curves generated from three independent replicates (*n* = 3) at each tested concentration. *NA* No activity detected at the tested screening concentrations

^**^p < 0.01 vs. positive control

^##^*p* < 0.01 vs. compound 4

The α-glucosidase inhibitory activity of the isolated compounds is presented in Table 6. Among the isolated compounds, only compound 4 exhibited weak inhibitory activity (IC₅₀ = 708.3 ± 1.025 µM; *p* < 0.0001 vs. positive control). Nevertheless, compound 4 was the most potent inhibitor of *α*-amylase enzyme activity, with an IC_50_ value of 19.32 ± 1.046 µM (*p* < 0.0001 vs. compounds 1, 2 and 3, Table 6).

## Discussion

*p*-Hydroxybenzoic acid (2), apigenin-7-*O*-*β*-ᴅ-glucopyranoside (3), and luteolin (4) were isolated and identified in the *B. tomentosa* EtOAc fraction using NMR analysis. Notably, the isolated compounds have not been previously isolated from *B. tomentosa* aerial parts, with apigenin-7-*O*-*β*-ᴅ-glucopyranoside (3) being previously isolated from the seed coat of *B. tomentosa* [39], whereas isorhamnetin and its derivatives have been reported to be present in the subgenus *Bauhinia* [2, 38]. Compounds 2–4 were further detected in the *B. tomentosa* EtOAc fraction using LC-QTOF-MS/MS analysis.

In 2024, 59 metabolites in *B. madagascariensis* and 66 in *B. purpurea* were identified in methanol extracts [40]. Moreover, 90 metabolites were identified in the aerial parts of *B. forficata*, *B. variegata*, *B. variegata* var. candida, and *B. galpinii* via LC-QTOF-MS [9]. Fatty acids, phenolics, flavonoids, and miscellaneous compounds were the main isolated compounds. All the structures were sufficiently ionized in negative mode with higher sensitivity, as expected.

To the best of our knowledge, no previous studies to date have investigated the differences in the antidiabetic efficacy of different *B. tomentosa* fractions; thus, this target was the focus of the present study in a trial to disclose the most effective fraction to optimize biological activity. In addition, an attempt was made to investigate the possible mechanisms by which different fractions could exert their beneficial effects. Finally, isolation and identification of the active compounds in the most active fraction, based on the results of serum glucose levels, was made in order to disclose the compound/s responsible for activity, and to further examine the potential mechanisms underlying the observed activity.

The results revealed that nicotinamide + STZ injection resulted in stable hyperglycemia, as well as slight but insignificant hypoinsulinemia. These findings are consistent with earlier studies [24, 41] and can be explained by the protective effect of nicotinamide against the diabetogenic action of STZ, which prevents massive pancreatic *β*-cell damage after STZ administration. Developed by Masiello et al. (1998), this model was designed to produce a state of partial insulin deficiency similar to that observed in T2D [24]. Thus, the nicotinamide + STZ model is an important tool for the pharmacological investigation of new insulinotropic agents [42]. The rapid and easy combined model was first described by Masiello et al. (1998) [24]. They reported that it produces a non-insulin-dependent, insulin-deficient T2D model that lacks overt insulin resistance [43]. The absence of pronounced insulin resistance in this model was also confirmed by Chao and coworkers [44].

The results of the present study revealed that treatment with different fractions of *B. tomentosa* for 14 days effectively reduced serum glucose levels in nicotinamide + STZ-induced T2D rats. The effect was most pronounced with the EtOAc fraction, followed by the DCM fraction, whereas the reduction was not significant in the rats treated with the aqueous fraction. Similarly, the serum insulin levels were significantly increased with the EtOAc and DCM fractions, when compared to untreated T2D group. These findings coincide with the previously well-documented antidiabetic activity of the *Bauhinia* genus, including *B. tomentosa* [25]. Furthermore, the insulinotropic effect of different *Bauhinia species* has been previously reported [25, 45–47], and this could be one of the possible mechanisms by which the EtOAc and DCM fractions exert their observed blood-glucose-lowering effect.

A non-significant reduction in GLUT4 levels in the skeletal muscle tissue homogenates of the nicotinamide + STZ-injected rats was observed when compared to the normal control group, a finding consistent with Chao and coworkers [44] Likewise, treatment with the different *B. tomentosa* fractions did not significantly increase total muscle GLUT4 expression compared with that in diabetic rats. GLUT4 plays a crucial role in regulating insulin-mediated glucose homeostasis and contributes to the pathogenesis of insulin-resistant metabolic diseases [48]. Although the EtOAc and DCM fractions significantly reduced serum glucose, this effect was not accompanied by a significant increase in total muscle GLUT4 expression. These findings suggest that the glucose-lowering effect of *B. tomentosa* may involve mechanisms other than increased GLUT4 expression, such as increased insulin secretion or the modulation of alternative glucose-regulating pathways. Furthermore, since the present study assessed total GLUT4 levels rather than its translocation to the plasma membrane, changes in GLUT4 trafficking cannot be excluded and warrant further investigation. Whether a longer treatment duration is required to manifest detectable changes in total GLUT4 expression also warrants further investigation.

Notably, the EtOAc fraction exhibited significantly greater glucose-lowering activity compared to the other *B. tomentosa* fractions. However, neither the serum insulin levels nor the muscle GLUT4 expression showed a significant difference among the different fractions. Similarly, the effects of the aqueous and DCM fractions did not differ significantly with respect to any of the measured parameters. Owing to this superior glucose-lowering activity, the EtOAc fraction was considered to possess the highest relative antidiabetic activity at the tested dose and treatment duration. Consequently, this fraction was selected as the most promising candidate for subsequent bioguided isolation, structural characterization, and in vitro mechanistic evaluation of its bioactive compounds.

In our study, the isolated compounds showed significant in vitro antioxidant activity. Antioxidants are well known to protect pancreatic beta-cells against oxidative stress-induced apoptosis and hence maintain their function. Consequently, antioxidant compounds may play a role in protection against diabetic-related complications [49]. This effect could contribute to the antidiabetic activity of the extract, as well as its potential effects on T2D, and was particularly observed with 4. As an important and possible antidiabetic mechanism, the *α*-glucosidase inhibitory activity of the isolated compounds was tested. Acarbose, a drug that targets the *α*-glucosidase enzyme, is capable of ameliorating hyperglycemia, particularly postprandial hyperglycemia, by inhibiting the intestinal absorption of carbohydrates [50, 51]. In line with this, voglibose, an *α*-glucosidase inhibitor, reduces oxidative stress generation in parallel with the reduction in postprandial hyperglycemia and hyperlipidemia in obese T2D patients [52]. Notably, in the current work, compound 4 was able to produce detectable (measurable) *α*-glucosidase inhibitory activity. Thus, this mechanism may only slightly contribute to the activity of the extract and is unlikely to represent its primary mode of action.

An additional crucial target in regulating blood glucose levels through the digestion of carbohydrates is *α*-amylase. *α*-Amylase has been employed as a drug target to inhibit postprandial hyperglycemia in diabetic patients. Luteolin (4) was the most potent inhibitor of this enzyme activity. This could also represent another possible mechanism of the antidiabetic activity of the extract. However, the exact full mechanism of glucose-lowering activity remains to be fully elucidated.

The metabolic profile of the EtOAc fraction contains several phenolic compounds, mainly flavonoids and flavonoid glycosides. Flavonoids are a group of naturally occurring polyphenolic secondary metabolites that have been reported to exhibit a wide range of pharmacological properties, most notably antidiabetic effects. For example, herbacetin inhibits fructose-1,6-bisphosphatase, reducing hepatic gluconeogenesis, and improves glucose metabolism in insulin-resistant cells in an insulin-resistant human hepatocellular carcinoma cell line (HepG2 cells) [53].

Scutellarein, 6-hydroxyapigenin-7-*O*-*β*- ᴅ -glucopyranoside, 6-hydroxyluteolin-7-*O*-β- ᴅ -glucopyranoside, 6-hydroxyapigenin-7-*O*-(6-*O*-feruloyl)-*β*- ᴅ -glucopyranoside, 6-hydroxyluteolin-7-*O*-(6-*O*-feruloyl)-*β*-ᴅ-glucopyranoside, and 6-hydroxyluteolin were also found to exhibit potent α-glucosidase inhibitory activity (92% inhibition at a concentration of 500 µM), with an IC_50_ value of 10 µM [54].

Quercetin and kaempferol inhibit glucose transporter activity and increase glucose uptake by activating the AMPK signalling pathway in skeletal muscle cells. Moreover, they inhibit *α*-glucosidase, protect pancreatic *β*-cells from damage caused by hyperglycemia-induced *β*-cell toxicity, and support their regeneration [55].

Eid and coworkers showed that quercetin could stimulate AMPK and increase GLUT4 translocation at a concentration of 50 µM after 18 h of treatment of cultured rat L6 skeletal muscle cells [56]. Moreover, quercetin aglycone could enhance glucose uptake in muscle cells. Similarly, quercetin has divergent effects on insulin-mediated GLUT4 translocation in adipocytes under basal and insulin-resistant conditions, depending on its regulation of AMPK activity [57].

Isorhamnetin, also known as 3’-methoxy quercetin, was also detected. Quercetin is methylated by the UDP-dependent glycosyltransferase, which transfers a methyl group to the ring and adds a glycoside function to isorhamnetin. In 2025, Li and coauthors reported that isorhamnetin exerts hypoglycemic effects while supporting *β*-cell integrity by activating the PI3K/AKT pathway and tempering the COX-2–mediated lipid mediator pathway in a streptozotocin (STZ)-induced model of type 1 diabetes [58]. Moreover, in a study examining the antidiabetic properties of quercetin and isorhamnetin, they promoted GLUT4 translocation to the plasma membrane in L6 myotubes via distinct mechanisms without altering GLUT4 expression at physiological concentrations. Furthermore, isorhamnetin activated the JAK-STAT signalling pathway at 1 nM and 10 nM, allowing the induction of glucose transporter translocation [59].

Although the isolated compounds demonstrated promising in vitro antioxidant and enzyme inhibitory activities, these findings represent preliminary evidence. Consequently, further investigations utilizing cell-based models, followed by in vivo studies and detailed mechanistic analyses, are needed to elucidate the underlying molecular mechanisms and validate the therapeutic potential of these compounds.

The observed antidiabetic activity of the isolated compounds is largely consistent with previous reports. For example, luteolin has been widely reported to play an important role in diabetes management through the inhibition of glucose absorption in the intestine, the promotion of glucose absorption and utilization by tissues, and the inhibition of the oxidative stress response and inflammation [60]. Consistent with these mechanisms, luteolin has been shown to reduce blood glucose levels, significantly improve oral glucose tolerance, and decrease insulin resistance [61]. In addition to these effects, luteolin promoted *β*-cell autophagy and protected *β*-cells from lipotoxicity-induced apoptosis, thus preserving *β*-cell function [62]. Apigenin-7-*O*-*β*-ᴅ-glucopyranoside can improve insulin resistance and regulate carbohydrate absorption and digestion. In addition, glycosylation improves water solubility and enhances the stability of the compound, thus contributing to great health benefits [63]. Interestingly, isorhamnetin (the core nucleus of compound 1) has been previously reported to exhibit antidiabetic activity. It can reduce glucose levels, ameliorate oxidative status, suppress inflammation, and regulate lipid metabolism [59]. Although previous studies have reported the antidiabetic activity of *p*-hydroxybenzoic acid [64], no significant activity was observed in the present study. Such differences may result from variations in experimental conditions or tested concentrations. Additional studies are needed to further clarify its antidiabetic potential.

A limitation of the present study is the absence of a standard antidiabetic drug as a positive control. The present work was designed as a bioassay-guided fractionation study aimed at identifying the most active fraction of *B. tomentosa* for subsequent phytochemical investigation. Although the primary aim was to compare the relative efficacy of the different *B. tomentosa* fractions, future studies should include established antidiabetic agents, such as metformin or acarbose, to facilitate direct pharmacological comparison.

Taken together, the isolated compounds, particularly luteolin (4), could be potential targets/ candidates in the treatment of T2D, with an expected well-documented safety profile that is known for *B. tomentosa.* The isolation and further identification of active compounds can yield promising agents for the treatment of diabetes and protection against T2D-related complications.

## Conclusions

The EtOAc fraction of *B. tomentosa* exhibited the most pronounced antidiabetic activity among the tested fractions in the in vitro assay. Thus, it was subjected to LC-QTOF-MS/MS analysis, which authenticated the metabolite fingerprint of *B. tomentosa* species, providing comparative data that aligns with the chemical profiles of phylogenetically linked species. In addition, a bioactivity-guided investigation of this fraction led to the isolation of four compounds (1—4). Compound 1, isorhamnetin-3-*O*-*α*-ʟ-rhamnoside-7-*O*-*α-*ʟ-3-methyl rhamnoside, was identified as a new compound. The antidiabetic effects of the EtOAc fraction could be attributed, at least in part, to its insulinotropic effect, antioxidant effect, and inhibition of *α*-amylase activity. The isolated compounds demonstrated varying antidiabetic activities in in vitro assays, indicating their potential as promising antidiabetic agents. Among the tested compounds, luteolin (4) exhibited the highest antidiabetic activity, whereas *p*-hydroxybenzoic acid (2) showed no detectable antidiabetic activity under the experimental conditions. However, the present findings are limited by the use of in vitro assays on isolated compounds. Therefore, further molecular and in vivo studies are needed to clarify the underlying mechanisms and to validate the potential of these metabolites as antidiabetic agents.

## Supplementary Information

Supplementary Material 1.

## Data Availability

The datasets used or analysed during the current study are available from the corresponding author upon reasonable request.

## Abbreviations

ANOVA: Analysis of variance
CC: Column chromatography
^13^C NMR: Carbon nuclear magnetic resonance
1D and 2D: Dimension
DEPT: Distortionless Enhancement by Polarization Transfer
DCM: Dichloromethane
DMSO: Dimethyl sulfoxide
DPPH: 2, 2-Diphenyl-1-picrylhydrazyl
ELISA: Enzyme-linked immunosorbent assay
ESI: Electron spray ionization
EtOAc: Ethyl acetate
GLUT4: Glucose transporter 4
^1^H-^1^H COSY: Correlated Spectroscopy
HMBC: Heteronuclear Multiple Bond Correlation
^1^H NMR: Proton nuclear magnetic resonance
HRP: Horseradish Peroxidase
IP: Intraperitoneal
LC-QTOF-MS/MS: Liquid Chromatography-Quadrupole Time-of-Flight Tandem Mass Spectrometry
M: Measured mass
MeOH: Methanol
m/z: Mass-to-charge ratio
MS: Mass spectrometry
Na_2_CO_3_: Sodium carbonate
NC: Normal control
NMR: Nuclear Magnetic Resonance
PBS: Phosphate-buffered saline
RT: Retention time
SE: Standard error
STZ: Streptozotocin
T2D: Type 2 diabetes
