# Supplementary material for: Phytochemical and antidiabetic evaluation of Bauhinia tomentosa L. aerial parts
Source: BMC Complement Med Ther. 2026 Aug 1;26:235. doi: 10.1186/s12906-026-05484-2 (PMC13430811; doi:10.1186/s12906-026-05484-2)
Supplement: Supplementary file 1 — Supplementary Material 1. [file 12906_2026_5484_MOESM1_ESM.docx]

**Supplementary materials**

**Scheme. 1 Extraction and fractionation of *B. tomentosa* aerial parts.**

**NMR data of the isolated compounds:**

**Compound 1**

**Fig. 1 ^1^H NMR spectrum of compound 1**

**Fig. 2 DEPT Q 135 spectrum of compound 1**

**Fig. 3 HSQC spectrum of compound 1**

**Fig. 4 HMBC spectrum of compound 1**

**Fig. 5 ^1^H ^1^H COSY spectrum of compound 1**

**
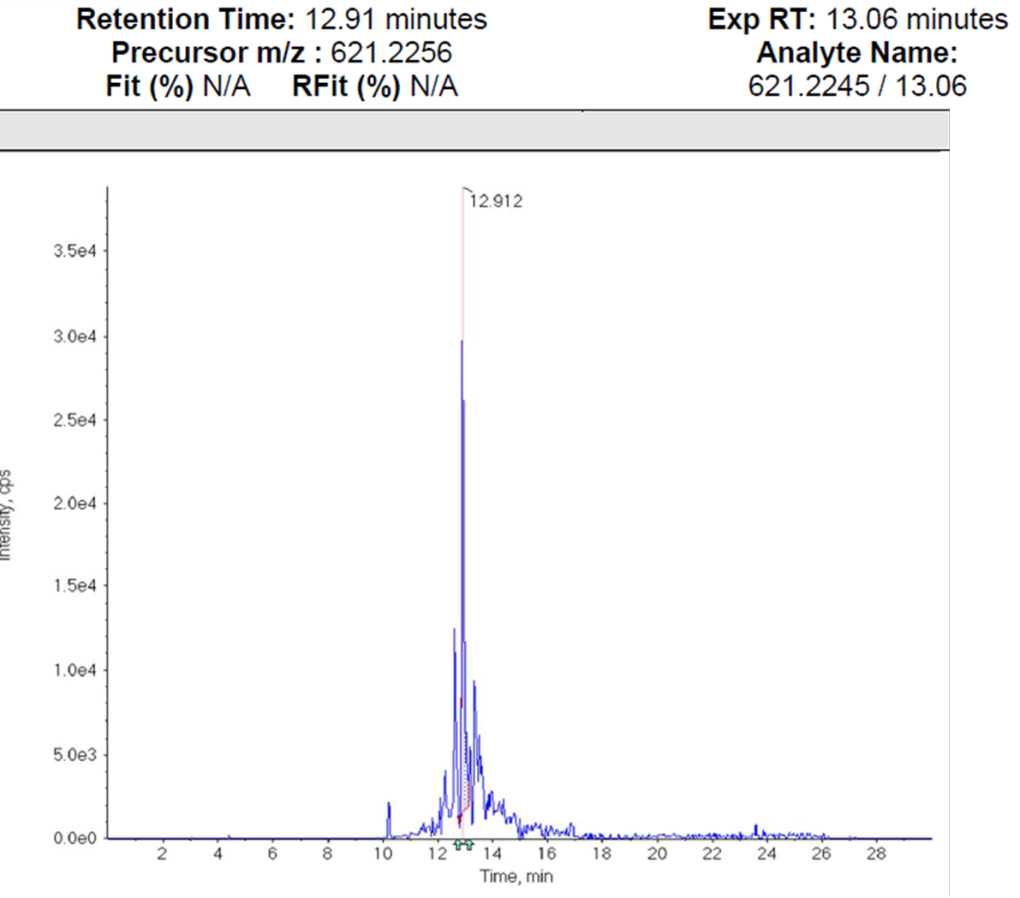
**

**
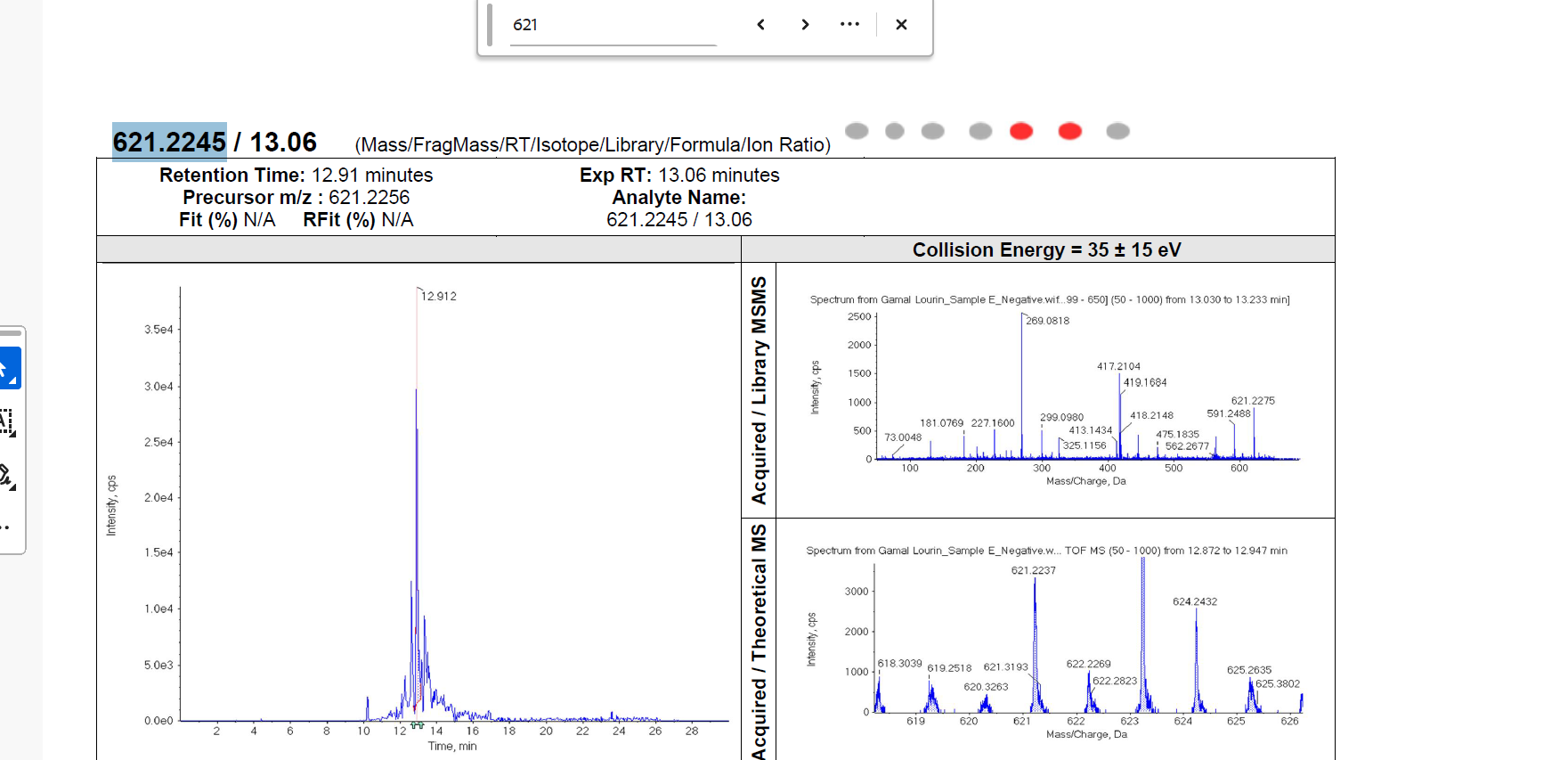
(a)**

**(b)**

**Fig. 6 (a) Mass spectrum of compound 1 [M-H] at 621.2256 m/z eluted at 12.91 minutes. (b) MS/MS fragmentation spectrum** **of compound 1 compared to the theoretical fragmentation spectrum in the library at 35±15 eV collision energy.**

**
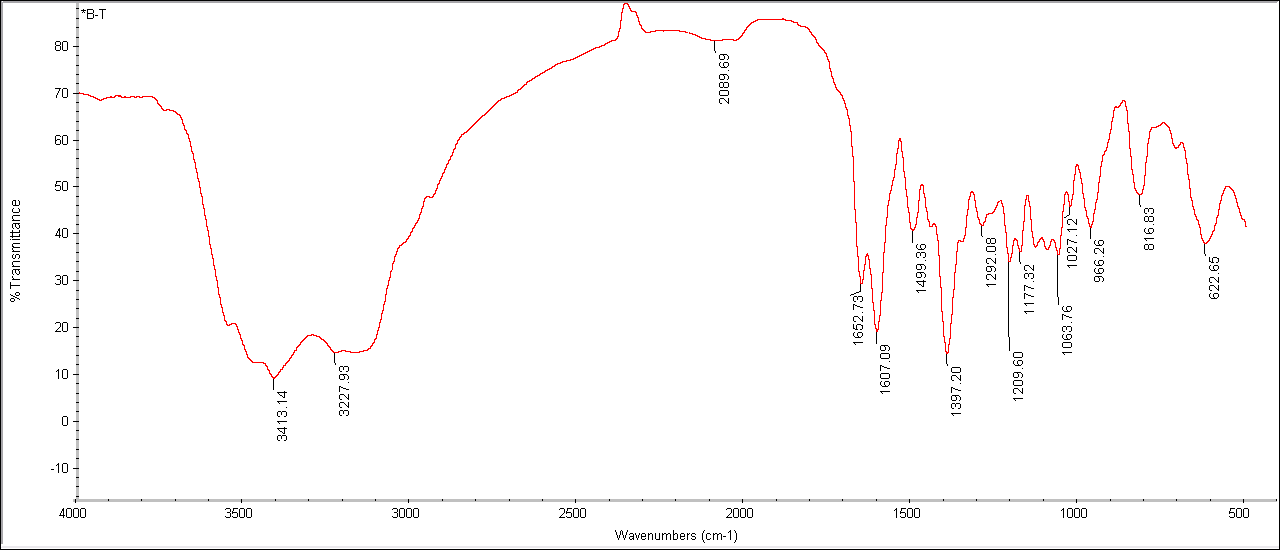
**

**Fig. 7 IR data of compound 1**

**Compound 2**

**Fig. 8 ^1^H NMR spectrum of compound 2**

**Compound 3**

**Fig. 9 ^1^H NMR spectrum of compound 3**

**Compound 4**

**Fig. 10 ^1^H NMR spectrum of compound 4**

**Fig. 11 DEPT Q 135 spectrum of compound 4**
